# Cystoscopy and Ureterscopy, with Exhibit of Instruments and Reports of Cases

**Published:** 1896-12

**Authors:** J. W. Long

**Affiliations:** Richmond, Va.; Professor of Diseases of Women, Medical College of Virginia


### CYSTOSCOPY AND URETERSCOPY, WITH EXHIBIT OF INSTRUMENTS AND REPORTS OF CASES.

By J. W. LONG, M. D., RICHMOND, VA.,

Professor of Diseases of Women, Medical College of Virginia.

Only recently has the bladder received the attention it deserves from gynecologists. Up to the last few years it was difficult to explore that viscera to determine the condition of its inner surface, and more difficult to explore the ureters.

Prior to the invention of the instruments I am about to exhibit, we were obliged to depend upon the dilatation of the urethra by a very crude method; indeed, stretching by the fingers, which often brought a permanent incontinence, is within the recollection of most of us. To explore the bladder was almost impossible, because of collapse of its walls, and, as said before, exploration and treatment of the ureters was attended with even more difficulty. For many years distension of the bladder with water and fishing around for the mouths of the ureters was our only resource. Not until Kelly accidentally struck upon the method to be described could we satisfactorily treat cystic and ureteral troubles. The method depends on—first, distension of the bladder with air, which a few years ago was thought to be exceedingly dangerous; and second, instruments whereby we may have a direct view of the inside of the bladder and the mouths of the ureters.

The instruments I show you are first, sixteen dilators, graded according to their measurement by the metric system. Numbers 10 to 14 are those usually employed. It is surprising to note the amount of stretching the female urethra can stand.

The second instrument is the calibrator, so called because it was originally intended to measure the size of the urethra. It is cone-shaped and graduated, and is now used n place of the original dilators.

At an early stage in the work it was found that the external meatus was the point of greatest resistance in dilating the urethra.

The third instrument is the cystoscope (made in various sizes), consisting of a straight tube flanged at one extremity, and having, projecting at an angle from near the same extremity, a handle. Fitting in this is an obdurator. After the urethra is dilated, the cystoscope is forced in by a gentle rotatory movement, and when the obdurator is withdrawn air rushes in, distending the bladder. I have suggested to Dr. Kelly that the cystoscope might, to advantage, be made shorter and with a more flaring flange.

The position the patient was made to assume at first was the dorsal, with hips much elevated. I have devised a pelvis elevator for this purpose. The bladder is first catheterized, the urethra dilated, the cystoscope introduced, and the residual urine drawn off by a suction apparatus, but it is almost a matter of impossibility to keep the bladder entirely clear of urine. A better posture is the knee-chest, because in it the residual urine gravitates from the floor of the bladder to the most dependent part, and the suction apparatus may be dispensed with. After exploring the entire inner surface of the bladder, which can be seen perfectly, it is easy to find the mouths of the ureters. Any kind of clear, reflected light can be used with a head mirror.

Through the cystoscope can be introduced the urethral searcher, which is a small sound with the handle bent at an obtuse angle. At first this is a difficult procedure, but practice makes it easy. I tried for eighteen months to catheterize the ureters, and had about decided that it was one of the things I could not do, when I suddenly hit upon it, and can now introduce the ureteral catheter in a few seconds.

The original ureteral catheter was devised by Simon. Pawlik modified it, and Kelly perfected the Pawlik instrument.

With a delicate pair of mouse-tooth forceps it is not at all difficult to pick a piece of cotton, stone, etc., from the bladder, or, with an applicator, to make an application to its walls.

Whalebone bougies are employed, first, to determine whether or not the ureters are patulous; second, to protect them during hysterectomy.

The vesical balloon, devised by Dr. Clark, consists of a fine rubber bag, with a stem of rubber tubing. It is used for the treatment of chronic vesical catarrh, where there are marked changes in the bladder walls, and which are not amenable to treatment by ordinary applications. He employs it covered with a ten per cent. gelatole of ichthyol, melting at the temperature of body. The balloon is buttered with the gelatole, rolled up, grasped in a pair of special forceps, introduced through the cystoscope, and distended with air, the medicine thereby reaching every part of the inner surface of the bladder. To prevent injury or rupture of the bladder, it is important to learn first how far the balloon should be distended.

By means of the cystoscope it has been discovered that many of the so-called cases of cystitis, especially in young women, are not general inflammations of the vesical mucosa, but hyperæmia or, at most, limited inflammations, and many of them are easily and rapidly cured by mild solutions of nitrate of silver, or any other astringent, applied through the cystoscope. I shall cite only one or two cases, to demonstrate its efficiency:

CASE I. was that of a young woman, married, who, for years, had had cystic trouble. Under cocaine anesthesia, the cystoscope being introdued and the area of inflammation determined, three or four applications of nitrate of silver sufficed to cure.

CASE II., a young woman whose tubes and ovaries had been removed two years before I saw her, for probably ovaro-salpingitis. After the operation, ventral hernia occurred, and she came for treatment. Inquiry revealed more suffering from the bladder than from the hernia, and brought to light the fact that for thirteen years she had been the victim of cystic trouble. There was very little pus in the urine, but a slow, irritating, aggravating cystitis was present. Application of nitrate of silver made it worse. Then Dr. Clark's method was employed. This, at first, was painful. Four applications, at intervals of three days, were made, and now there is complete cure. I afterward relieved the hernia, which was as large as a peck measure, by operation.

I wish it clearly understood that I claim nothing original in this work. Dr. Kelly should be credited with a large part of the recent advances. Knowing that I was working along the same lines, he has urged me to report my results, which heretofore I have not done, but which I hope to do more extensively at no distant day.

*200 West Grace Street.*

